# P61 SARS-CoV-2 vaccine safety in adolescents with inflammatory rheumatic and musculoskeletal diseases and adults with juvenile idiopathic arthritis: data from the EULAR COVAX physician-reported registry

**DOI:** 10.1093/rap/rkac067.061

**Published:** 2022-09-28

**Authors:** Saskia Lawson-Tovey, Pedro Machado, Anja Strangfeld, Elsa Mateus, Laure Gossec, Loreto Carmona, Bernd Raffeiner, Inita Bulina, Daniel Clemente, Julija Zepa, Ana Rodrigues, Xavier Mariette, Kimme Hyrich

**Affiliations:** Centre for Genetics and Genomics Versus Arthritis, Centre for Musculoskeletal Research, The University of Manchester, Manchester, United Kingdom; National Institute of Health Research Manchester Biomedical Research Centre, Manchester University NHS Foundation Trust, Manchester Academic Health Science Centre, Manchester, United Kingdom; Centre for Rheumatology and Department of Neuromuscular Diseases, University College London, London, United Kingdom; National Institute for Health Research University College London Hospitals Biomedical Research Centre, University College London Hospitals NHS Foundation Trust, London, United Kingdom; Department of Rheumatology, Northwick Park Hospital, London North West University Healthcare NHS Trust, London, United Kingdom; German Rheumatism Research Center (DRFZ Berlin), Epidemiology Unit, Berlin, Germany; Portuguese League Against Rheumatic Diseases (LPCDR), Lisbon, Portugal; European League Against Rheumatism (EULAR) Standing Committee of People with Arthritis/Rheumatism in Europe (PARE), Kilchberg, Switzerland; Sorbonne Université, INSERM, Institut Pierre Louis d'Epidémiologie et de Santé Publique, Paris, France; Pitié-Salpêtrière Hospital, AP-HP, Rheumatology Department, Paris, France; Instituto de Salud Musculoesquelética, Madrid, Spain; Department of Rheumatology, Central Hospital of Bolzano, Bolzano, Italy; Pauls Stradins Clinical University Hospital, Riga, Latvia; Riga Stradins University, Riga, Latvia; Hospital Niño Jesús, Madrid, Spain; Pauls Stradins Clinical University Hospital, Riga, Latvia; Riga Stradins University, Riga, Latvia; Reuma.pt, Sociedade Portuguesa de Reumatologia, Lisbon, Portugal; EpiDoC unit, CEDOC, Nova Medical School, Lisbon, Portugal; Rheumatology Unit, Hospital dos Lusíadas, Lisbon, Portugal; Department of Rheumatology, Université Paris-Saclay, Assistance Publique-Hôpitaux de Paris, Hôpital Bicêtre, INSERM UMR1184, Le Kremlin Bicêtre, France; Centre for Epidemiology Versus Arthritis, Centre for Musculoskeletal Research, The University of Manchester, Manchester, United Kingdom; National Institute of Health Research Manchester Biomedical Research Centre, Manchester University NHS Foundation Trust, Manchester Academic Health Science Centre, Manchester, United Kingdom

## Abstract

**Introduction/Background:**

There is a lack of data on Severe Acute Respiratory Syndrome Coronavirus 2 (SARS-CoV-2) vaccination safety in children and young people (CYP) with rheumatic and musculoskeletal diseases (RMDs) as they were excluded from initial vaccine trials. Vaccination guidance is based on data from adults with or CYP without RMDs.

**Description/Method:**

Our objective was to describe the safety of SARS-COV-2 vaccination in adolescents with inflammatory RMDs and adults with JIA. We described patient characteristics, flares, and adverse events in adolescent cases under 18 with inflammatory RMDs and adult cases aged 18 or above with JIA submitted to the European Alliance of Associations for Rheumatology (EULAR) COVAX registry.

**Discussion/Results:**

Thirty-six adolescent cases were reported from 4 countries, mostly female (58%) with JIA (42%: 28% non-systemic JIA, 14% systemic JIA) and a median age of 15 [IQR: 14.5, 17]. Most were in remission (64%) or had minimal (22%) disease activity at the time of vaccination. Over half of the adolescent group (56%) reported early reactogenic-like AEs. One mild polyarthralgia flare and one serious AE of special interest (malaise) were reported. No CYP reported SARS-CoV-2 infection post-vaccination. No cases of paediatric inflammatory multi-system syndrome or myocarditis adverse events were reported.

Seventy-four adult JIA cases were reported from 11 countries; 73% were female with a median age of 26 [IQR: 23, 31]. Eight-five percent had ns-JIA and 15% had s-JIA. Almost two thirds (62%) reported early reactogenic-like AEs and two flares were reported (mild polyarthralgia and moderate uveitis). No serious AEs of special interest were reported among adults with JIA. Three 20-30 year old females were diagnosed with SARS-CoV-2 post-vaccination; all fully recovered.

**Key learning points/Conclusion:**

In this observational registry dataset, SARS-CoV-2 vaccines appeared safe in adolescents with RMDs and adults with JIA, with a low frequency of disease flares, serious AEs, and SARS-CoV-2 re-infection seen in both populations.

